# Challenges of Continuous Wave EPR of Broad Signals—The Ferritin Case

**DOI:** 10.1007/s00723-024-01719-y

**Published:** 2024-10-26

**Authors:** Fabio Seiji Otsuka, Maria Concepción García Otaduy, Otaciro Rangel Nascimento, Carlos Ernesto Garrido Salmon, Martina Huber

**Affiliations:** 1 InBrain Lab, Department of Physics, Faculty of Philosophy, Sciences and Letters of Ribeirão Preto (FFCLRP), University of São Paulo, Ribeirão Preto, Brazil; 2 Huygens-Kamerlingh Onnes Laboratorium, Leiden Institute of Physics, Leiden University, Leiden, The Netherlands; 3 Laboratory of Medical Investigation (LIM44), Clinic’s Hospital of the Medicine School of the University of São Paulo (HCFMUSP), University of São Paulo, São Paulo, Brazil; 4 São Carlos Institute of Physics (IFSC), University of São Paulo, São Carlos, Brazil; 5 Department of Medical Imaging, Hematology and Clinical Oncology, Faculty of Medicine of Ribeirão Preto (FMRP), University of São Paulo, Ribeirão Preto, Brazil

## Abstract

The study of continuous wave (cw) electron paramagnetic resonance (EPR) spectra still poses a challenge for very broad signals, especially when the spectrum extends over a large part of the accessible field range. The difficulties derive from instrumental challenges, because of insufficient modulation depth and the need to apply measurement conditions that enhance cavity background. The biggest problem, however, is how to define a baseline such that spectral distortions are minimized. Conventional methods rely on a suitable choice of points outside the range of the signal of interest to perform a polynomial interpolation. These methods are effective in most cases where the signal of interest comprises only a narrow range of magnetic field (narrow features). In this study, a novel method of baseline correction for broad signals is proposed and compared to conventional methods. It takes into account that there are only few anchor points for the baseline. The method is applied to the signal of the iron-storage protein ferritin. The ferritin signal is a broad band that extends from zero to 0.8 T. An approach is developed by which this broad signal is analyzed reliably. The method is also extended to the case where the broad signal is superimposed on narrow signals and enables to extract the parameters of both types of signals in a fitting pipeline.

**Supplementary Information:**

The online version contains supplementary material available at 10.1007/s00723-024-01719-y.

## Introduction

**Fig. 1:**
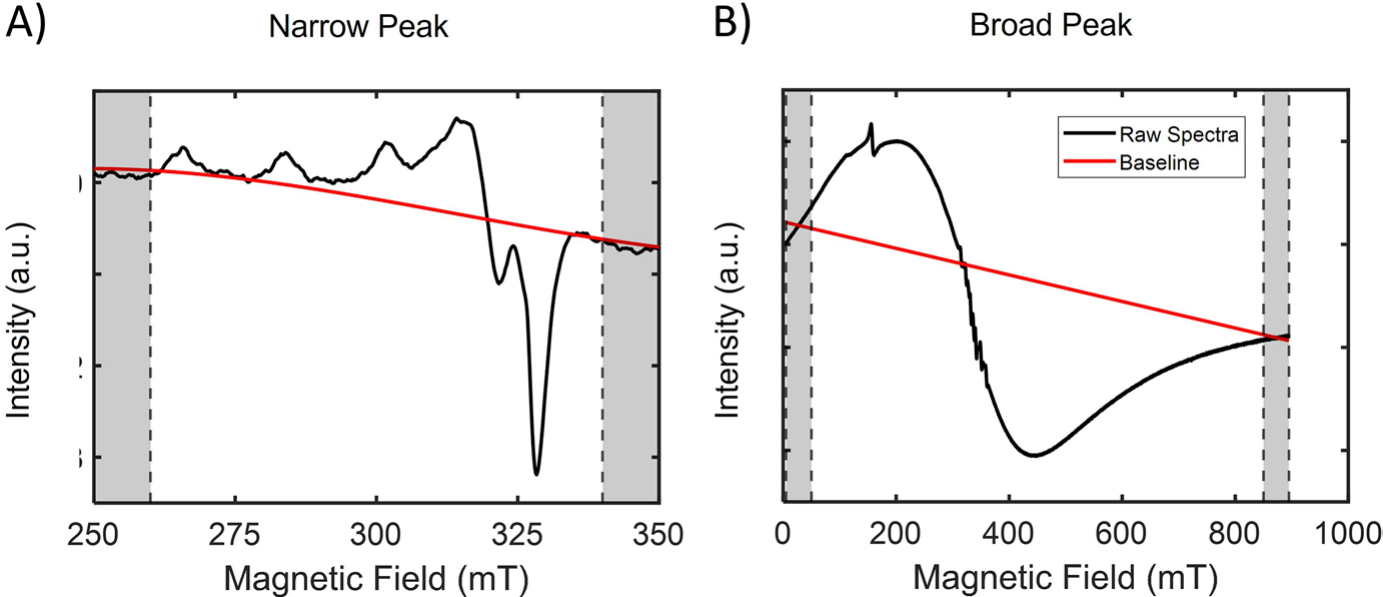
Comparison of spectra with a well-defined narrow peak (left) and a very broad peak (right) which covers the entire field range. Shaded areas show points taken for baseline determination. Left: Copper signal from a human brain sample (HuBrain) recorded at 60 K, a second-order polynomial was used for baseline estimation; right: spectrum from human liver ferritin (HuLiFt) recorded at 60 K, a first-order polynomial was used for baseline estimation

Continuous wave (cw) electron paramagnetic resonance (EPR) spectroscopy faces multiple challenges when very broad EPR signals are investigated. The situation is illustrated for the cw EPR spectrum of ferritin, shown in Fig. 1b, on which we will focus in this study. Many other systems, from material sciences to biology, can have broad EPR signals, so the approach is relevant for diverse fields of research.

From the measurement point of view, broad signals, such as those of ferritin, are challenging: Signal intensity is limited and measurement conditions can enhance background signals above what is observed for the more common narrow-line signals. Particularly challenging is that the signal spreads over most of the accessible field range, requiring special approaches for baseline correction. For narrow features, the background is obtained using a function, as shown in red in Fig. 1a that interpolates between regions of the spectrum in which the background is clearly defined (shaded areas in Fig. 1a). However, in the case of the ferritin signal, Fig. 1b, there is no obvious field region in which to define a baseline, and it is likely that in the shaded area at low magnetic field (B_0_) values, i.e., at B_0_ from zero to 50 mT, the broad signal still has intensity, and therefore this region should not be used for anchoring the baseline. In addition, there is no part in the spectrum in which the quality of the baseline could be checked, unlike the case of the narrow-line signal, see above and Fig. 1a. Therefore, for broad signals, there is the risk that the baseline chosen affects the signal shape, and causes spectral distortions. Given that the lineshape is of critical importance to interpret the spin state of any compound with a broad line, the question of how the raw data of broad-line spectra are treated becomes an important issue. Here we describe an approach that was developed to minimize spectral distortions to avoid lineshape distortions, illustrated for the example of the EPR spectrum of ferritin.

The interest and the motivation to study the ferritin EPR signal derives from the biological significance of the protein [1–3]. Ferritin is the main iron-storage protein in organisms [4, 5]. It stores iron ions in a spherical core that measures approximately eight nanometers in diameter, and that is surrounded by a protein shell. The mineral form of the iron oxide in the core is still under debate. Catalytic sites in the protein shell channel iron into and out of the core as needed by the cell.

Recently, ferritin from human liver has been investigated by EPR [6]. The spectrum of this isolated ferritin, shown in Fig. 1b, has the broad signal at *g* = 2.01 that covers the magnetic field range from 0 to almost 1 T in the 9 GHz EPR spectrum. It also contains a narrow signal around *g* = 4.3 that is assigned to high-spin (S = 5/2) Fe(III) in a rhombic local symmetry. In ferritin, the *g* = 4.3 feature is ascribed to iron centers in the protein shell, but other sources, such as Fe(III) ions, bound to the surface of the protein cannot be fully excluded.

The broad signal, due to the ferritin core, reflects the spin state of the approx. 2000 iron ions of the core. Proper quantitative evaluation of its characteristics is difficult and literature results have focused on qualitative approaches, using nano-particle-derived expressions [7–9]. Additional information comes from changes in the spectra as a function of the temperature, revealing that ferritin is superparamagnetic at higher temperatures, and upon decreasing the temperature, it exhibits an antiferromagnetic behavior, with a blocking temperature, derived from EPR, around 100 K [9].

Ultimately, analysis of EPR spectra in terms of the spin-Hamiltonian parameters is usually done by comparing the spectrum to a simulation that derives from a spin-system model that is chosen according to the spectral properties, lineshape and intensity, and their changes with temperature. Experimental spectra that have broad signals have few resolved features, and therefore can be simulated with a large number of different spin systems. Under these conditions, spin systems need to be chosen on physical reasoning and automatic fitting routines must be used with great caution, as models with many free parameters require more spectral information than found in the broad-line signal. In Ref. [6], it was shown that a minimum of two components was required to simulate the spectra of ferritin, and that fitting of the parameters for the two-component case results in unphysical results.

In the present account, we describe a strategy for baseline correction of broad EPR spectra, using the signal of ferritin (Ft) from different sources as an example. We describe two cases, one where the broad signal dominates the spectra at most temperatures, here the isolated HuLiFt was chosen, and one, derived from human brain tissue (HuBrain), where, besides the broad ferritin signal, several narrow signals are observed. We compare two baseline-correction methods, the one traditionally used for narrow (BC1) and one modified to be suitable for broad lines (BC2). In addition, we test if the giant-spin model [10] can be applied to the broad signal in spectra from human brain tissue, presented in Ref. [11]. We show that, both, the giant-spin model and the baseline approach used in Ref. [6], but described in more detail here, can be incorporated into the fitting pipeline used in Ref. [11].

## Materials and Methods

### Parameters Used in EPR Experiments

HuLiFt cw EPR spectra were recorded with a 9 GHz EPR spectrometer (BRUKER, Elexys system E680) with a helium flow cryostat for temperature control and an Oxford temperature unit connected to a thermocouple for temperature readout. HuBrain cw EPR spectra were recorded with a 9 GHz EPR spectrometer (VARIAN, E109) with a helium flow cryostat for temperature control and an Oxford temperature unit connected to a thermocouple for temperature readout. Experimental parameters: modulation amplitude, time constant, and field range are depicted in Table 1 for both samples.

**Table 1:** EPR parameters used for the HuLiFt and HuBrain

	HuLiFt	HuBrain
Microwave frequency (GHz)	9.50	9.11
Power (mW)	20	0.5
Modulation amplitude (mT)	2.9	0.8
Modulation frequency (kHz)	90	100
Time constant (ms)	10.24	29.30
Field range (mT)	5—895	50—650

### Considerations for Measurement Conditions and Preprocessing

For broad lines, the sensitivity is limited by the hardware, as the maximal modulation amplitude of the spectrometer is usually far below the one optimal for the broad signal, which would be approximately 10–20% of the peak-to-peak linewidth of the spectrum. As usually for such signals, longitudinal relaxation times (T_1_) sufficiently short, high microwave powers can be used to increase the signal amplitude; however, high powers also enhance background signals of the microwave resonator.

To analyze the spectra, first the resonator background is subtracted. This often additionally requires subtracting a linear background, as high modulation amplitudes enhance the background slope.

### Detailed Description of the Baseline-Correction Method Applicable for Broad Lines (BC2)

To avoid any spectral distortions, while also guaranteeing that a baseline is reached on the absorption spectra (i.e., the first integral of the EPR spectra), a new approach was developed using first-order polynomials while looking at the integral spectrum behavior (BC2 method).

This approach assumes that the first integral of the spectrum behaves like an absorption spectrum, which means that the first integral of the EPR signal returns to zero at the high-field side of the absorption line. Alternatively, this means that the second integral of the spectra should reaches a plateau.

To fit the spectrum in this criterion, an iterative algorithm was developed, termed here BC2 method. The algorithm starts by creating an initial linear baseline using a given number of end points of the spectrum. The choice of a linear baseline was made to avoid introducing any spectral distortions that higher order polynomials could introduce. The selected points at the end of the spectra must be chosen such that no visible signal is observed at that region.

Then two conditions are imposed:

1. The high field region of the linear baseline should be locked.
2. The second integral must reach a plateau at the end, which means that the absorption spectrum (first integral) has reached a baseline as well.

The first condition is imposed at the beginning of the code by fixing the end point of the baseline as the mean value of the last points of the spectrum. The second condition is reached by iteratively changing the slope of the linear baseline, while verifying how the slope of the last points on the second integral varies.

To change the slope, while accounting for Condition 1, the amplitude at the low field of the linear baseline is changed by a defined step at each iteration. Then a linear baseline is calculated and subtracted from the spectra and a second integral spectrum is obtained. The code then evaluates the resulting second integral spectrum by looking at the slope of the high-field points that were used in Condition 1. This can be achieved by applying a linear fitting to the high-field points. If this slope is higher than a defined value (tolerance level), then the iteration continues, otherwise the iteration stops, and the last linear baseline is given.

To increase the robustness of the algorithm, at the end of each iterative step, the algorithm evaluates if the modulus of the slope is decreasing, otherwise it changes the direction in which the slope is varied.

### Analysis of EPR Spectra, Workflow

Each spectrum was baseline corrected using the two different approaches, the BC1 and BC2 method. BC1: To perform a baseline correction, points at the beginning and at the end of the spectra were set for the interpolation, and different polynomials from first to fourth order were compared. As for BC2, only the points at the end of the spectra were considered. The choice of points was as follows:

1. Points at the beginning: Points below 25 mT for HuLiFt and below 50 mT for HuBrain
2. Points at the end: Points above 860 mT for HuLiFt and above 650 mT for HuBrain

Baseline-corrected spectra by each method were visually compared for a qualitative evaluation.

For a quantitative comparison, second integral values of the baseline corrected spectra were calculated and plotted against temperature. In addition, a relative difference was calculated by considering the BC2 method as a reference. No other processing was applied to the spectra to avoid introducing other variables into the results.

A relative difference was calculated using the BC2 method as a reference. Then the percentual difference was calculated as:

1 $$RD=100\frac{\left({BC1}_{n}-BC2\right)}{BC2} ,$$

where $RD$ is the relative difference in percentage, and ${BC1}_{n}$ represents the BC1 method with the n-th order polynomial (n = 1 to 4).

### Processing Pipeline for EPR Spectra

To demonstrate the feasibility of spectra fitting for broad features (HuLiFt), as well as for more complex systems (HuBrain), a fitting-pipeline recommendation is given.

All the fitting processing was performed using the EasySpin toolbox (version 5.2.35) in Matlab (version R2021a) using the ***pepper*** function.

Since for the HuBrain spectra, the narrow peaks dominate the signal, the suggestion is to start by fitting them before baseline correction and broad-signal analysis. It was found that for the Cu(II) peaks, narrowing the full spectral range to the field range where the Cu(II) signal is found is the best starting point. In this case, a polynomial fitting is enough to eliminate the baseline (Fig. 1a). As for the *g* = 4.3 signal, we used the literature values [12], by also narrowing the field range and applying a polynomial fitting to eliminate the baseline contribution in this field range. After subtraction of the narrow peaks from the original spectra, the broad feature can be more easily identified (at low temperatures). An alternative approach such as a low-pass/high-pass filter approach seems less promising, as some narrow features, such as the Cu(II) signal (Fig. 1 a), have a complex lineshape that may not be completely removed by such an approach, and therefore was not attempted.

For the fitting of the broad feature from both HuLiFt and HuBrain, the ferritin model proposed in Ref [6]. was implemented. Briefly, the model consists of two components that overlap at a g-value of *g* = 2.01, but with different zero-field splitting parameters D and anisotropic broadening (HStrain).

## Results

A typical spectrum that we analyze here is shown in Fig. 1b. We describe the approaches to perform a baseline correction in a way that results in reliable lineshapes. Also, we describe how to perform the analysis in cases where broad and narrow signals overlap. Measurement conditions and considerations for pre-treatment of the data are described in Materials and methods.

**Fig. 2:**
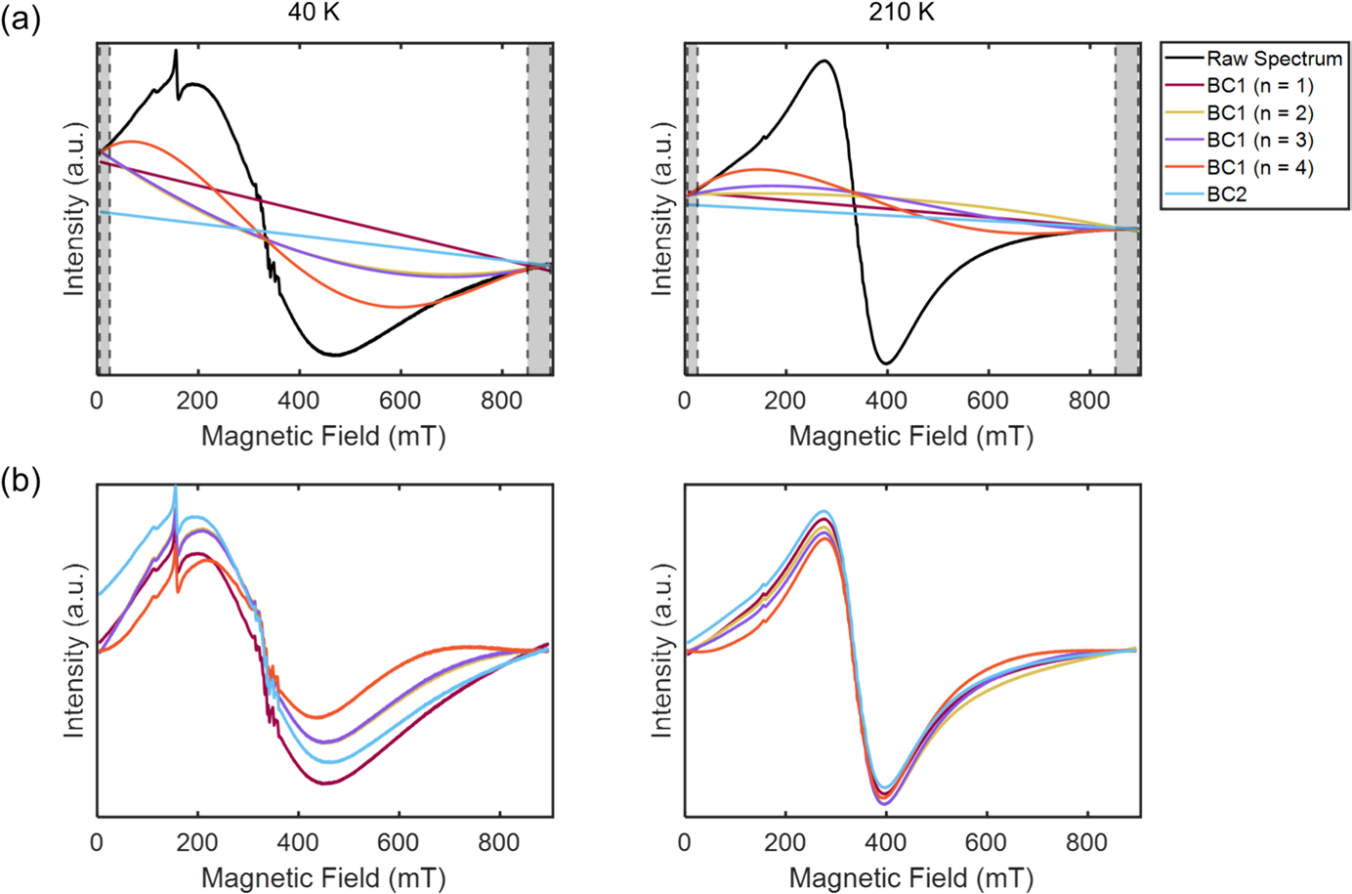
EPR spectra of a broad EPR spectrum for two temperatures (40 K, left; 210 K, right) and different baseline models. **a** Baselines determined by different approaches, shaded areas delimited by dashed lines indicate the points that were used for the baseline determination; for BC2, only the points at high field were used. **b** Baseline-corrected spectra for each baseline-correction approach. Note that for the spectrum at 40 K, **a** the background line BC1 (n = 2) line is not visible because it closely follows the BC3 (n = 3) line

Figure 2 illustrates the effect of the traditional method of baseline correction (BC1) on the lineshape of broad signals, using the spectra of HuLi ferritin at two representative temperatures as an example. The top two traces show the baselines resulting from interpolation between the spectral regions marked in grey (BC1, n = 1–4) and the bottom traces show the resulting spectra. The spectral lineshapes differ significantly, depending on the polynomial degree. The deviations are accentuated for the spectrum obtained at low temperature (40 K); yet in all cases, the spectral differences are so large that the interpretation of the lineshape would be affected. Experimentally, the proper degree of polynomial cannot be decided, which makes lineshape analysis impossible.

The distortions observed in BC1 (n > 1) suggest that a new approach is needed. It has to take into account that signal may not vanish in the low field (between 0 and 50 mT), and that regions in the spectrum devoid of signal are sparce. To account for these problems, we developed a different approach, addressed here as the BC2 method. The method uses a linear baseline and keeps the high-field region as an anchor for the baseline, but most importantly, it replaces the extrapolation to the low-field region by the requirement that the total cw-EPR spectrum must integrate to an absorption lineshape.

**Fig. 3:**
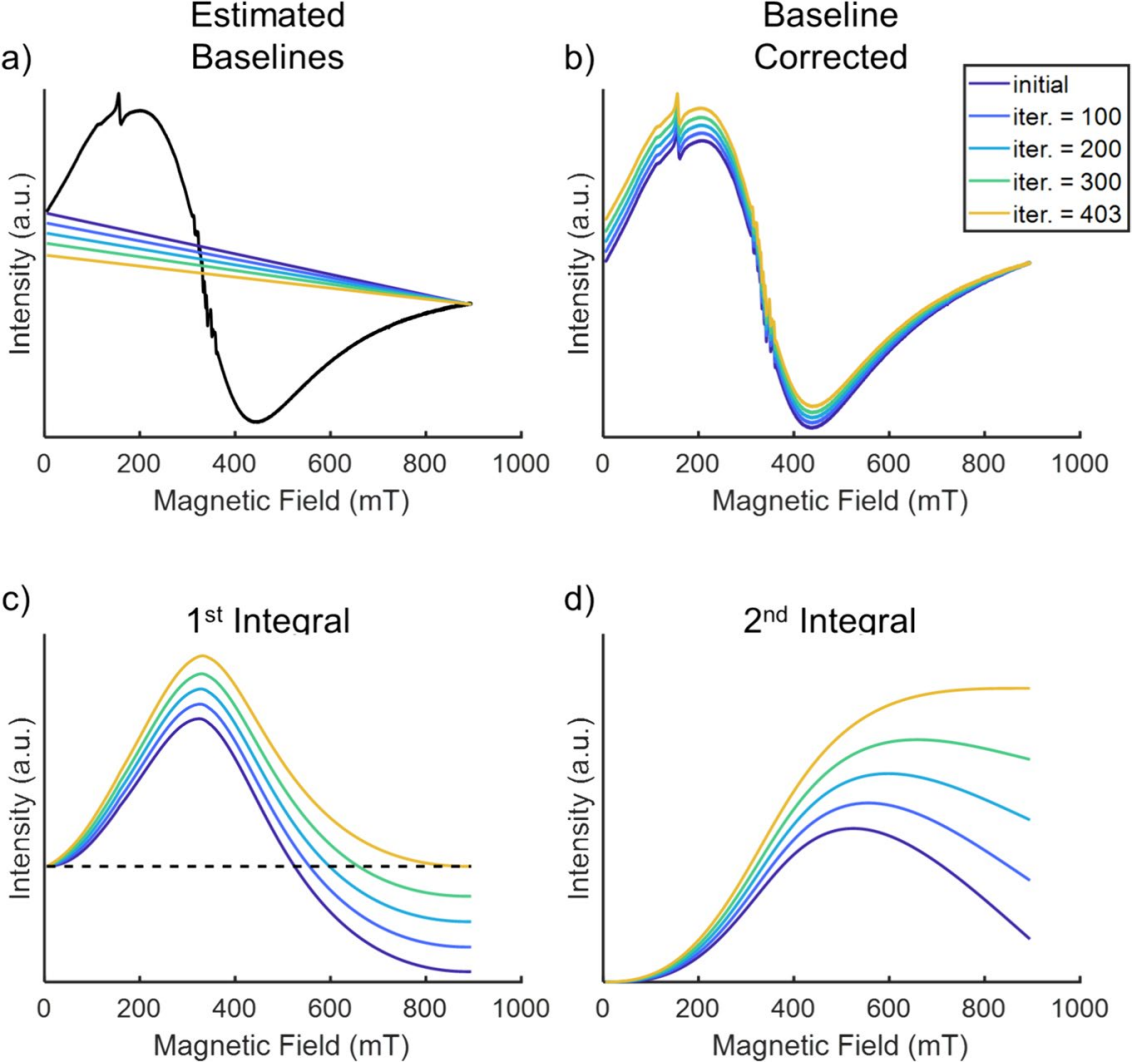
Illustration of the baseline-correction method BC2. Plots of the possible baselines (**a**), baseline-corrected spectra (**b**), first integral of the baseline-corrected spectra (**c**) and second integral of the baseline-corrected spectra (**d**) over some iterations of the BC2 method for the HuLiFt at 60 K. The black curve in (**a**) shows the raw spectrum, and the dashed line in (**c**) indicates the zero amplitude. Yellow line: Optimal baseline for experimental spectrum

Figure 3 shows the steps of the BC2 method. In Fig. 3a, the spectrum is shown along with four steps of baseline iteration with dark blue representing the starting baseline and yellow, the optimum baseline. For these steps, Fig. 3b shows the respective resulting spectra, shown in the same color code, and Fig. 3c the first and Fig. 3d the second integrals.

Technically, the following steps are performed: The slope of a linear baseline, anchored in the high-field area shown in gray shading in Fig. 2, is adjusted until the integral of the spectrum returns to baseline at high fields (above 860 mT, flat, amplitude zero shown in Fig. 3c. Alternatively, this means that the second integral ends in a horizontal line (Fig. 3d. The iterative procedure is described in Materials and methods in detail (BC2 method).

**Fig. 4:**
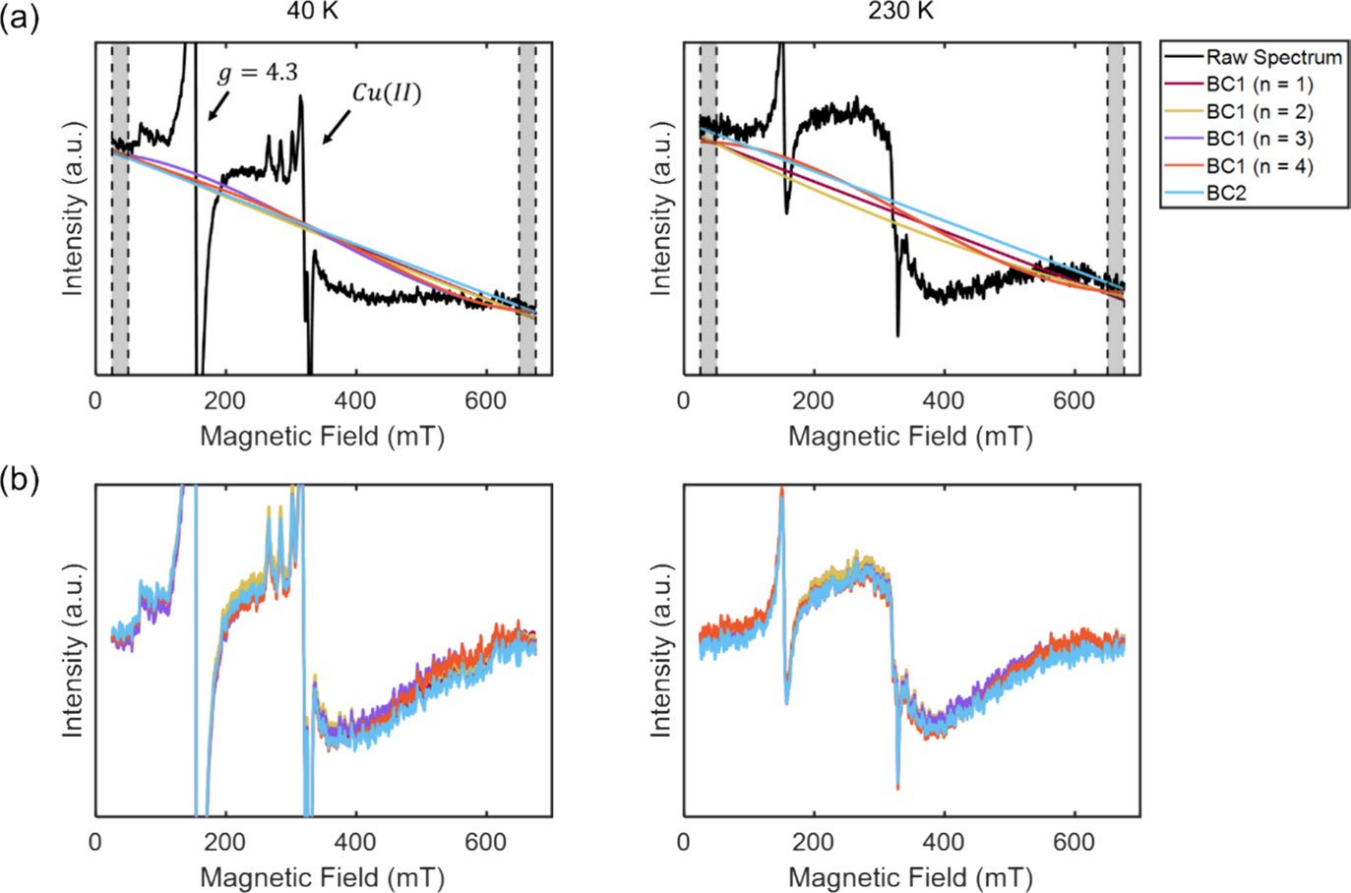
EPR spectra of HuBrain for two temperatures (40 K, left; 230 K, right) and different baseline models. **a** Baselines determined by different approaches, shaded areas delimited by dashed lines indicate the points that were used for the baseline determination; for BC2, only the points at high field were used. **b** Baseline-corrected spectra for each baseline-correction approach

Further considerations apply when the spectrum contains narrow lines in addition to the broad signal. The EPR spectra of brain material, shown in Fig. 4, are interesting and relevant examples. Besides a significant fraction of ferritin, also a mononuclear, high-spin, S = 5/2 Fe(III) signal, the so-called *g* = 4.3 signal, and a Cu(II) signal are observed (Fig. 4a). Figure 4 shows a typical spectrum of such a sample of lyophilized human brain tissue at different temperatures. For these samples, both the narrow and the broad features are of interest, so the analysis described in Ref. [11] takes both into account. Here we focus on extracting the broad signal. Figure 4 also shows different baseline-correction approaches applied to the HuBrain spectrum. It shows that, similar to the HuLiFt, also for the HuBrain, the BC1 method results in lineshape changes, especially for higher order polynomials, and therefore is not well-suited to obtain undistorted spectra.

**Fig. 5:**
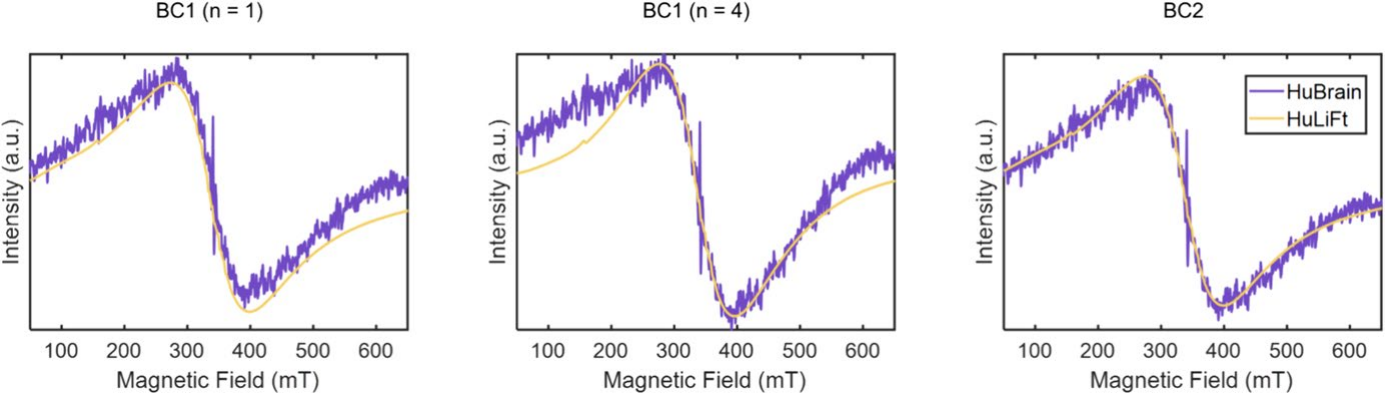
Baseline-corrected human brain (HuBrain) and human liver ferritin (HuLiFt) spectra at 190 K superimposed using different baseline-correction approaches. Spectra were scaled to match the peak-to-peak amplitude of the broad feature, and corrected for the microwave frequency. The *g* = 4.3 peak and copper signal were subtracted from the HuBrain spectrum for better visualization

To illustrate this, in Fig. 5, the methods of baseline subtraction are compared for two ferritin spectra of different origins. Spectra of HuLiFt and HuBrain were individually baseline corrected using the same method and superimposed. For a better visualization, narrow features were subtracted from the HuBrain spectra.

Assuming that both samples should have similar broad-line shapes, it can be seen that only the BC2 method showed a good consistency, where both spectra are in good agreement. BC1 (n = 4) showed significant distortions to the spectra, resulting in large disagreement of spectra.

**Fig. 6:**
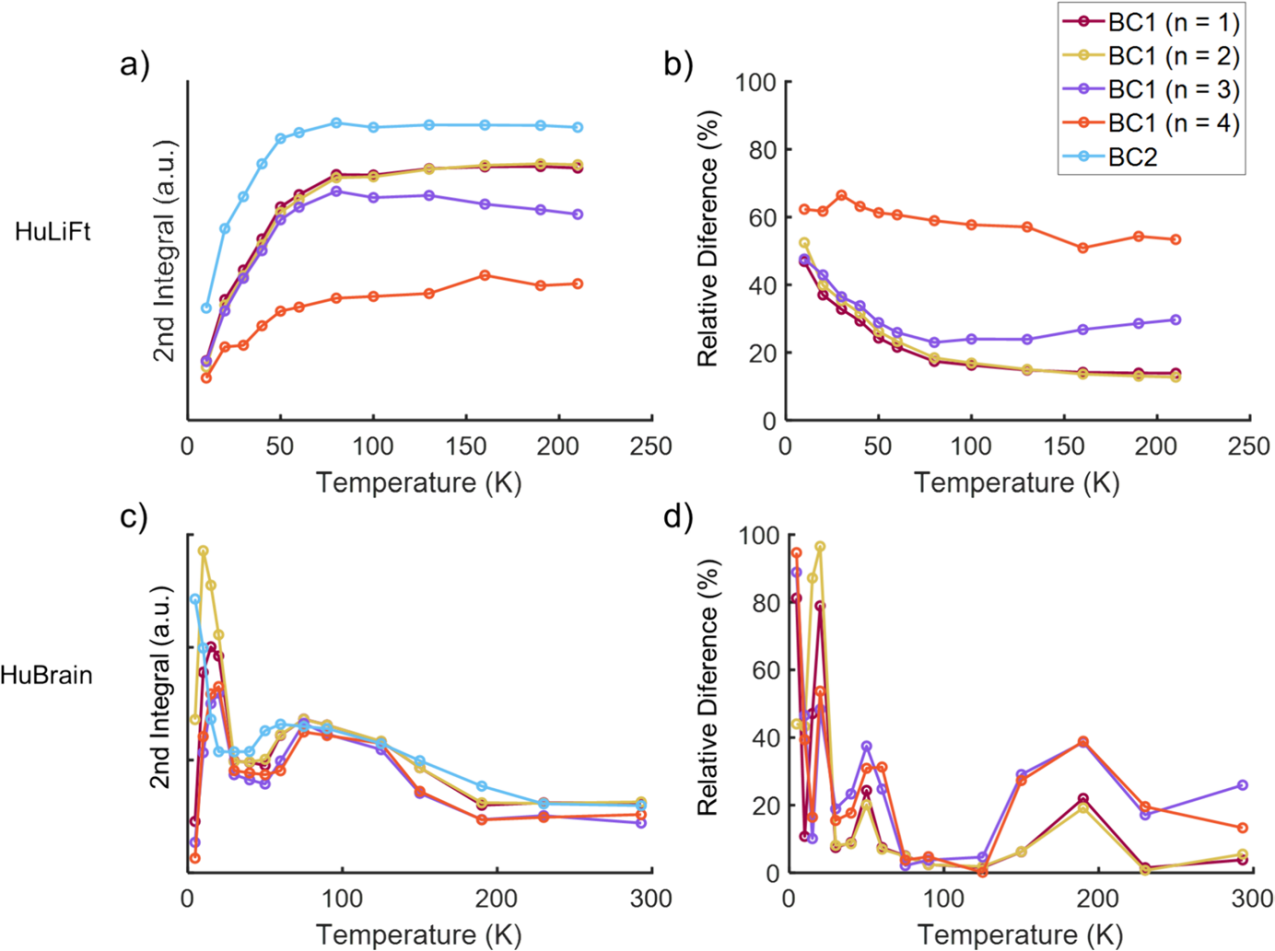
Comparison among different baseline approaches in HuLiFt (top) and HuBrain (bottom) samples for different temperatures and considering second integral values. Left: Absolute values. Right: Relative difference (%) considering the BC2 method as a reference

Another way to assess the effect of baseline correction over the entire spectrum is to map the double integral over the whole spectral range as a function of temperature and compare the impact of the different baseline-correction approaches. For the HuLiFt spectrum in Fig. 6, the signal intensity increases as the temperature is raised, demonstrating an antiferromagnetic behavior. For the HuBrain spectra, the intensity decreases as the temperature is raised, suggesting a paramagnetic behavior, which is reversed at a temperature of 40 K, when the intensity increases again, revealing an antiferromagnetic contribution.

The curves in Fig. 6a for the HuLiFt spectral intensity of BC1 (n = 3 and 4) differ from those of the lower polynomials and the BC2 method, for the HuBrain sample in Fig. 6c, all BC1 curves have a low intensity at 5 K, a trend not seen for the BC2 method. Assuming that the baseline treatment should not affect the trend of the signal intensity, the differences between the curves in Fig. 6 suggest that BC1 with higher polynomials is problematic. To illustrate this, in Fig. 6b and d, the relative difference of the spectral intensity, calculated using Eq. (1), is shown. The higher the relative difference, the stronger the impact of the baseline correction on the spectra.

Figure 6b shows the largest deviations for n = 3 and 4, yet all BC1-corrected spectra differ significantly from the BC2-corrected spectra at temperatures below 50 K.

Apart from the baseline determination, the quantitative interpretation of the EPR signals to obtain their spin-Hamiltonian parameters is required to understand which paramagnetic states these signals represent. This is performed by simulating or fitting the EPR signals with a spin-Hamiltonian approach. The narrow signals have characteristic features that enable to assign them to Cu(II) ions, mononuclear Fe(III) with a *g* = 4.3 feature, etc. An approach was suggested in Otsuka et al. (2022) [11] to perform this analysis on HuBrain samples. Here we summarize the approach briefly and describe the modifications made.

The suggested approach starts by fitting the narrow features individually (see Materials and methods). In Otsuka et al. 2021, all EPR signals were fitted after baseline correction. This does not work well with the approach used in the present account, because the BC2 background is affected by the presence of the narrow signals, especially at low temperatures. Therefore, to apply the BC2 method, it is necessary to subtract the narrow signals before applying a baseline correction by the BC2 method. To do so, the narrow features are simulated, and then subtracted from the broad signal.

Figure 1a illustrates how baseline correction, according to the BC1 method is done, for the example of the Cu(II) signals in the HuBrain sample. Then the narrow signals are fitted individually and subtracted from the raw spectra. Finally, the spectrum with the narrow features subtracted is used to determine the baseline with the BC2 method.

A brief account of the simulation of the narrow signals: For the Fe(III), the *g* = 4.3 signal is fitted; however, the starting parameters given in Table 2 yield a good agreement with the actual *g* = 4.3 feature, around 160 mT, so the fitting step is not strictly necessary. For the spectra in the 5 K to 20 K temperature range, deviations are seen around 60 mT and at 560 mT, suggesting that the parameters are not optimal yet.

**Table 2:** Starting parameters for the fitting of each peak of the HuLiFt and HuBrain spectra

		${g}_{x}$	${g}_{y}$	${g}_{z}$	${A}_{\perp } (MHz)$	${A}_{\| } (MHz)$	$D (MHz)$	$E (MHz)$
Fe(III) high-spin	HuLiFt	$1.805$	$1.982$	$2.010$	$-$	$-$	$20960$	$7017$
	HuBrain	$1.805$	$1.982$	$2.010$	$-$	$-$	$20960$	$7017$
Cu(II)	HuBrain	$2.038$	$2.038$	$2.220$	$34.63$	$559.37$	$-$	$-$
Ft.Sys1*	HuLiFt	$2.01$	$2.01$	$2.01$	$-$	$-$	$170$	$-$
	HuBrain	$2.01$	$2.01$	$2.01$	$-$	$-$	$130$	$-$
Ft.Sys2*	HuLiFt	$2.01$	$2.01$	$2.01$	$-$	$-$	$400$	$-$
	HuBrain	$2.01$	$2.01$	$2.01$	$-$	$-$	$170$	$-$

*For the broad feature (Ft.Sys1 and Ft.Sys2), the g-value was kept constant at 2.01 and the D and broadening parameters were varied at a fixed interval. Therefore, a set of simulations was performed instead of a fitting procedure

As for the Cu(II) signal, a reasonable fitting was obtained using the initial parameters given in Table 2. However, a closer inspection of this peak shows the possibility of two different copper species overlapping. In future studies, acquisitions of this region with higher SNR should be performed for better evaluation.

The broad signal, ascribed to the ferritin core, is more difficult to simulate because it has less defining features. We use the approach described in Ref. [6]. It consists of simulating the broad signal with two overlapping signals. To determine the simulation parameters, a fitting approach is bound to fail: A large number of free parameters, combined with the low spectral resolution of the broad signal, causes the fitting hypersurface to have many local minima. This means that different sets of parameters can give a good fit; however, they may not correspond to a reasonable physical interpretation. Therefore, instead of relying on the fitting procedure, a series of simulations, model simulations, was performed, resulting in suitable parameters for D, the zero-field splitting and HStrain, the Gaussian broadening. Due to computational limitations, a scaling approach based on the giant-spin model [10] is employed.

**Fig. 7:**
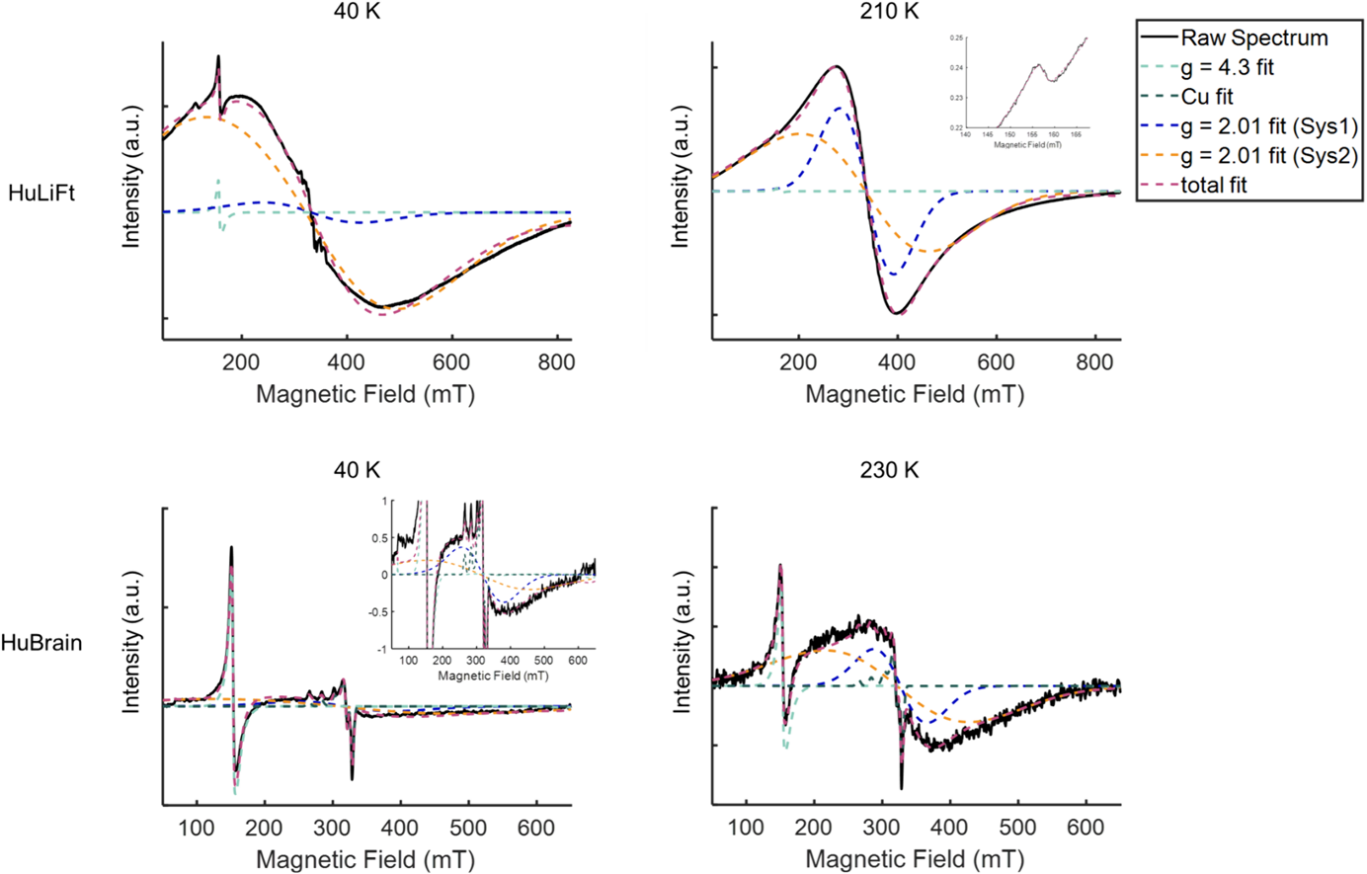
EPR signals of the HuBrain sample, showing the simulation approach. BC2-corrected raw spectra (black) and the corresponding fitted peaks (dashed lines) for different samples and temperatures. Top row: HuLiFt at 40 K (left) and 210 K (right), inset on the right graph depicts the *g* = 4.3 fitted peak; bottom row: HuBrain at 40 K (left) and 230 K (right), inset on the left graph depicts the *g* = 2.01 fitted peak

The simulation is conducted by choosing suitable parameters of D and the broadening parameter for each component and for their relative contribution to each spectrum. Next, these parameters were adjusted, until a visual inspection revealed a good agreement of the simulation with the experimental signal. This resulted in the simulations shown in Fig. 7 and in Supplementary Data (Figs. S1 and S2). The starting parameters for the broad signal simulations are given in Table 2. For the broad signal, the g-value was kept constant at *g* = 2.01. Then simulations were done using different D and HStrain parameters.

## Discussion

As described in the Introduction, with broad EPR signals, such as the one of ferritin shown in Fig. 1b, EPR faces multiple challenges. Since the modulation amplitude in continuous wave (cw) EPR, here 3 mT, is too low to reach optimum sensitivity for lines with a width of several tens of mT, the signals are weak; yet, modulation amplitudes at the limit of the instrumentation enhance cavity background artefacts that at lower modulation amplitudes are not critical. Also, high modulation amplitudes can cause a sloping, linear baseline contribution via Lorentz forces on the modulation coils. To increase the amplitude of signals, high microwave powers are used whenever possible, but they can also enhance unwanted cavity background signals, for example from impurities. And, finally, one has to take into consideration that signals can extend beyond the magnetic field (B_0_) sweep range of the spectrometer or have a non-vanishing amplitude at zero magnetic field, see Fig. 1b. All these features require that the regular preprocessing steps have to be adjusted and optimized to obtain reliable lineshapes. Next, the analysis of the signal in terms of the magnetic resonance parameters requires special care. In this study, we describe these problems and suggest approaches to solve them.

At first, we demonstrate that the standard approach for baseline correction, here referred to as the BC1 method, leads to inconsistencies that show up in lineshape changes depending on the order of the polynomial, see Fig. 2. The problem has two causes: First, as seen in Fig. 1b, the broad signal extends over almost the entire measurement range, so there are not many regions in the spectrum that can be defined as baseline. For narrow-line spectra, even if they extend over a wide field range, intermediate points, where no signal occurs, can usually be found, enabling to anchor the baseline at various regions of the spectra. The second reason is that, at zero field, the spectrum does not reach the baseline, so defining points in the low field part of the spectrum as baseline lead to errors in the lineshape.

We, therefore, present method BC2 that avoids the second cause and limits the correction to first order as the most conservative approach for baseline correction (see results). Figures 2 and 3 show the impact of the different baseline-correction approaches on the lineshape.

In addition, we also investigated the impact of the baseline correction on the overall spectrum by evaluating the second integral of the entire spectra as a function of the temperature (Fig. 6). For the pure ferritin spectra shown in Fig. 6a, an antiferromagnetic behavior is expected, see Ref. [6] and references therein, leading to a decrease of signal intensity toward lower temperatures. Although all baseline-correction methods show this overall trend, the relative deviation of the BC1 method with respect to the BC2 method (Fig. 6b suggests that the BC1 method introduces undesired artefacts, certainly when high polynomials (n = 3 or 4) are used. In conclusion, the BC2 method seems to be the method that is least likely to introduce artefacts in the lineshape and, therefore, is recommended.

Next, we address the case where the spectrum contains, in addition to the broad signal, also narrow signals, arising from other paramagnetic centers in the sample. In the HuLiFt spectra, these are signals at *g* = 4.3, around 160 mT, and around *g* = 2, around 300 mT (Fig. 1b). These signals do not interfere with the analysis, except for the very lowest temperatures, and therefore are not considered further. A different situation arises in the EPR spectra of HuBrain. [11]

In the HuBrain spectra, the narrow signals are more intense, and they are of interest, because they reflect other paramagnetic species in the brain. Besides, the amplitude of the signals is so large that they interfere with the analysis of the broad signal as performed above.

We show that to analyze all spectra of a temperature series of the HuBrain sample, it is better to first determine the lineshape of the narrow-line signals, by spectral simulation or fitting, subtract them from the raw spectrum, and then analyze the broad-line spectrum.

Once the spectra are appropriately pre-processed, spectral simulation of the broad signal is desired to obtain the magnetic parameters of the species responsible, in our case the ferritin core. In analogy to Ref. [6], we use the giant spin model (see results). We show that for the rather featureless signal of ferritin, a fitting approach does not work. Fitting algorithms invariably get trapped in local minima and yield physically unreasonable results. Therefore, a simulation approach, based on model spectra, is used. In the present study, we also show that fitting of the narrow signals in the HuBrain sample seems to work. A full interpretation of the parameters obtained for Cu(II) and mononuclear Fe(III) signals will be given in the future.

In summary, in this study, we show that the investigation of very broad EPR signals presents special challenges, and how they can be overcome. We specifically address the background problem and how to deal with eventually present narrow signals. We also describe how to analyze the broad signals. For the specific problem of the ferritin spectrum, either the isolated ferritin from human liver (HuLi) or the ferritin-containing brain (HuBrain) samples, we provide a processing pipeline that enables reliable processing of spectra taken as a function of the temperature. We are confident that this approach can be adapted to a wide range of broad-line EPR spectra, providing a method that enables tackling the specific challenges involved in analyzing such spectra.

## Conclusion

This work explores the effect of baseline correction on cw EPR spectra containing broad features while proposing a novel method for baseline correction that was shown to have an overall better performance in comparison to conventional methods for baseline correction, i.e., simple interpolation with polynomial functions. We show that with conventional methods, the resulting spectra present severe spectral distortions, which impact both qualitative and quantitative analysis of spectra with broad signals. Considering spectra in which a broad signal occurs in two different situations—HuLiFt and ferritin and other paramagnetic signals in lyophilized human brain—we show that the newly developed baseline-correction method results in spectra that are more consistent across temperatures and sources. Also, a method was developed to quantitatively analyze narrow and broad-line signals in a spectrum. Finally, a fitting pipeline was proposed and evaluated for both samples by implementing current literature approaches. The resulting pipeline resulted in a good agreement between the experimental data and the fitted data, while resulting in reasonable values for the evaluated spin systems.

## Supplementary Information

Below is the link to the electronic supplementary material.

Supplementary file1 (PDF 717 kb)

## Data Availability

Data is provided within the manuscript and supplementary information files.
