# Supplementary material for: Challenges of Continuous Wave EPR of Broad Signals—The Ferritin Case
Source: Appl Magn Reson. 2024 Oct 26;55(12):1605–20. doi: 10.1007/s00723-024-01719-y (PMC11582094; doi:10.1007/s00723-024-01719-y)
Supplement: Supplementary file 1 — Supplementary file1 (PDF 717 kb) [file 723_2024_1719_MOESM1_ESM.pdf]

Supplementary Data for

Challenges of Continuous Wave EPR of Broad Signals -The Ferritin Case

Fabio Seiji Otsuka[1], Maria Concepción García Otaduy[3], Otaciro Rangel Nascimento[4], Carlos Ernesto Garrido Salmon[1,5], Martina Huber[2]

[1]InBrain Lab, Department of Physics, Faculty of Philosophy, Sciences and Letters of Ribeirão Preto (FFCLRP), University of São Paulo, Ribeirão Preto, Brazil

[2]Huygens-Kamerlingh Onnes Laboratorium, Leiden Institute of Physics, Leiden University

[3]Laboratory of Medical Investigation (LIM44), Clinic's Hospital of the Medicine School of the University of São Paulo (HCFMUSP), University of São Paulo, São Paulo, Brazil

[4]São Carlos Institute of Physics (IFSC), University of São Paulo, São Carlos, Brazil

[5]Department of Medical Imaging, Hematology and Clinical Oncology, Faculty of Medicine of Ribeirão Preto, Ribeirão Preto, Brazil

Applied Magnetic Resonance

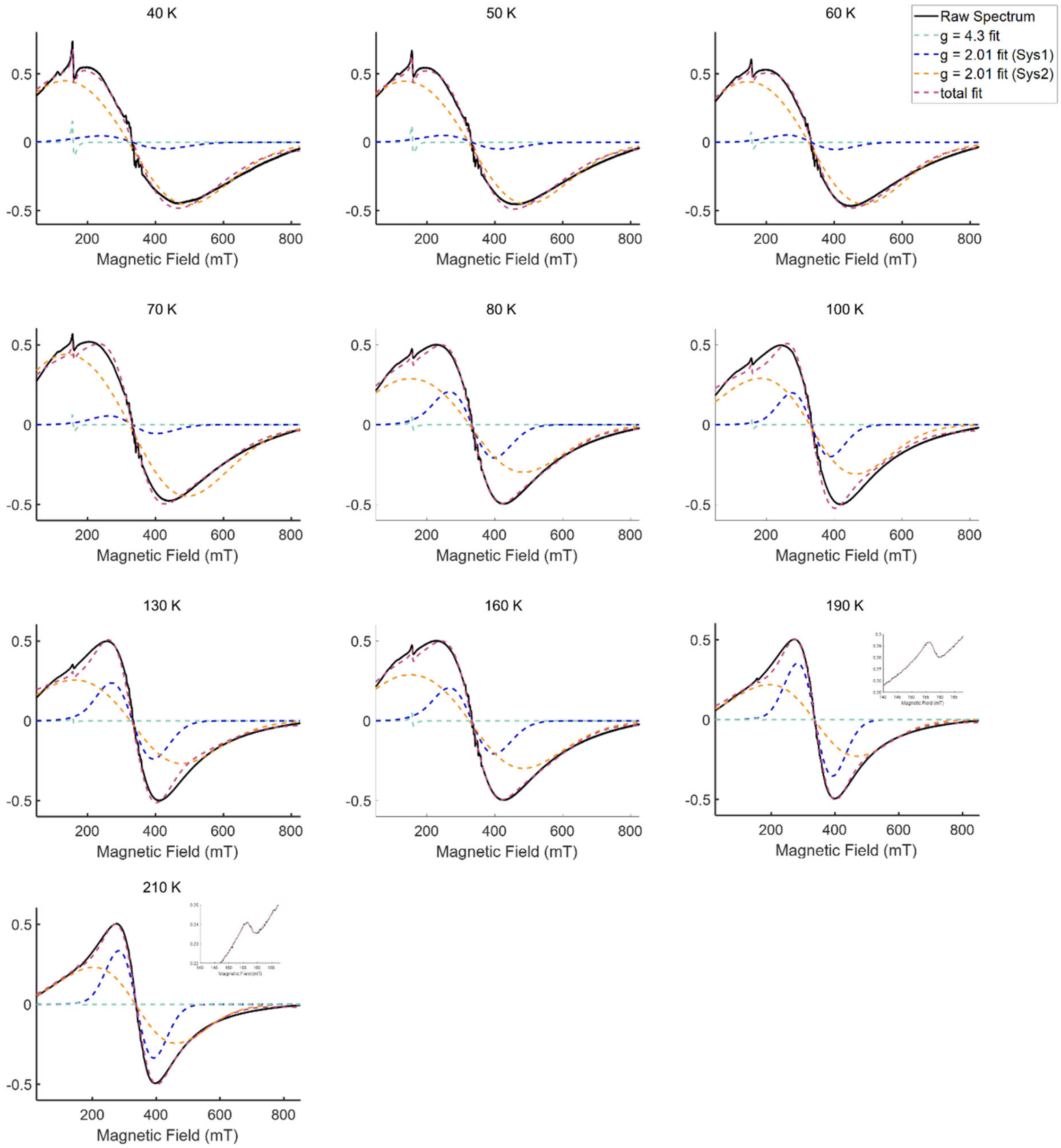

Figure S1 – Raw spectra (continuous line) and fitted spectra (dashed lines) for the HuLiFt spectra at different temperatures – experimental spectra from ref [6] main text.

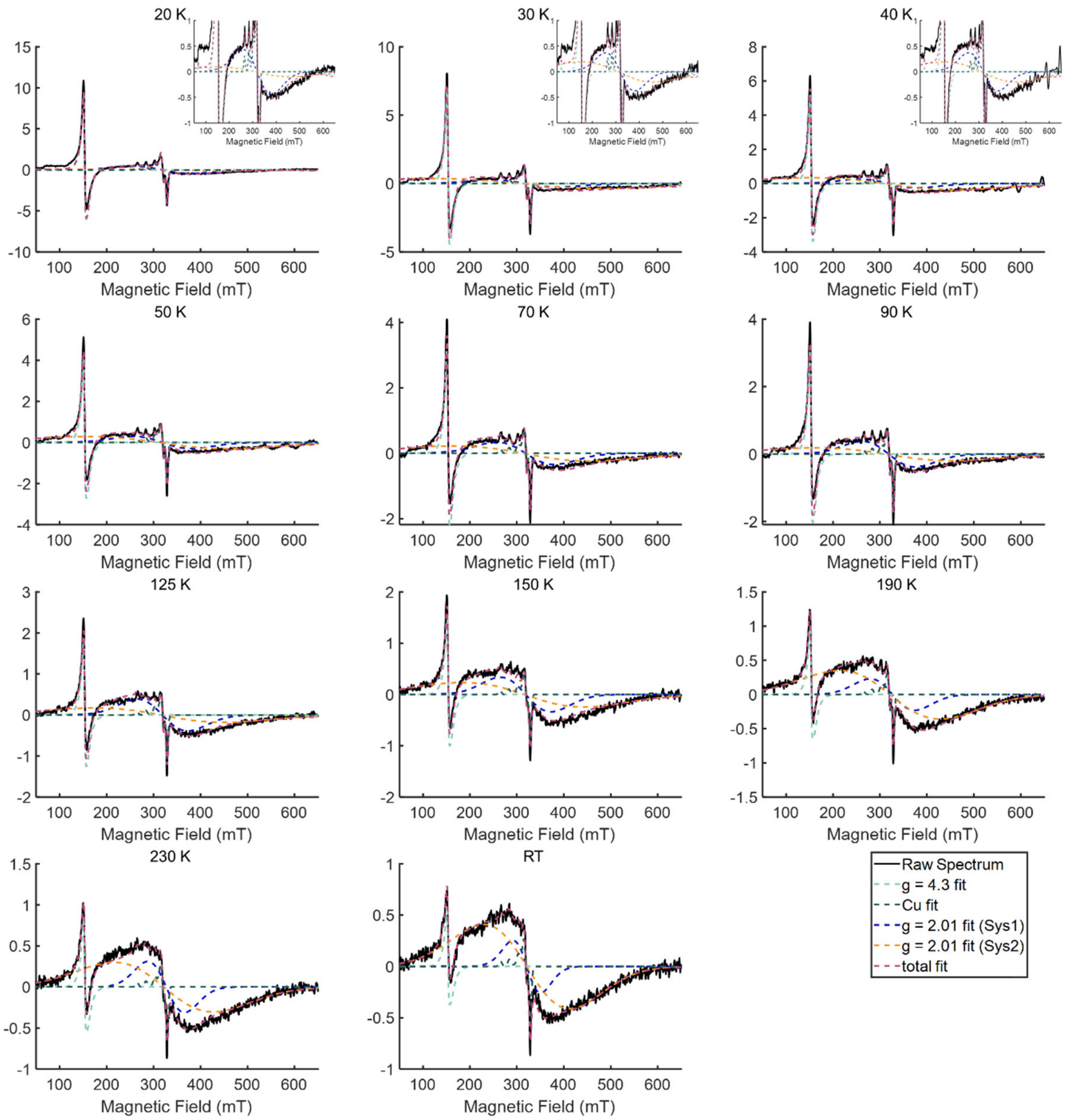

Figure S2 – Raw spectra (continuous line) and fitted spectra (dashed lines) for the HuBrain spectra at different temperatures.
